# Metagenomes from Late Pleistocene Ice Complex Sediments of the Siberian Arctic

**DOI:** 10.1128/MRA.01010-19

**Published:** 2019-10-24

**Authors:** Tatiana Vishnivetskaya, Elena Spirina, Lyubov Shmakova, Maria Tutukina, Zhou Li, Xiaofen Wu, Elizaveta Rivkina

**Affiliations:** a Institute of Physicochemical and Biological Problems in Soil Science, Russian Academy of Sciences, Pushchino, Russia; b University of Tennessee, Knoxville, Tennessee, USA; c Institute of Cell Biophysics, Russian Academy of Sciences, Pushchino, Russia; d Oak Ridge National Laboratory, Oak Ridge, Tennessee, USA; Georgia Institute of Technology

## Abstract

The late Pleistocene Ice Complex (also known as Yedoma) encompasses ice-rich permafrost formed when alluvial and/or aeolian sediments accumulated under cold climatic conditions. Three metagenomes obtained from Yedoma deposits continually frozen for periods up to 60,000 years are reported here.

## ANNOUNCEMENT

The late Pleistocene Ice Complex contains massive polygonal ice wedges and segregated ice (1) that formed during syngenetic deposition when freezing and ground ice accumulation occurred simultaneously with sedimentation (2). The Yedoma sediments are widely distributed in northeastern Siberia (3), representing important archives of paleoenvironmental conditions (4), and are vulnerable to thawing (5). These deposits contain about 10^15^ kg of organic carbon, with 10% of this carbon being decomposable (2). A release of this carbon due to microbial decomposition upon permafrost thaw may increase greenhouse gas (GHG) emissions to the atmosphere (5, 6). However, there remain substantial uncertainties associated with the microbial component of the Yedoma permafrost environment.

Yedoma deposits located on the Bykovskiy Peninsula (BP) and Omolon River (OR) mouth were frozen under cold, arid climatic conditions about 50,000 to 60,000 and 25,000 to 28,000 years ago (3, 4), respectively, while Yedoma deposits from the Gydanskiy Peninsula (GP) represent slightly saline, silty loams with peat intercalations and a permafrost age of 34,300 years (7). Samples collected from the BP (at a depth of 24.3 m) and the OR (at a depth of 26.5 m) had ∼1.1% total organic carbon, and methane was not detected (3, 4). On the other hand, the GP sample (from a depth of 4.5 m) had <1% total organic carbon and biogenic methane in concentrations up to 0.4 mmol kg^−1^ (7). Permafrost cores were obtained using a portable gasoline-powered drill that operates without a drilling fluid so as to ensure aseptic sampling (8). A peripheral portion of the extracted permafrost cores was removed with a sterile knife (9), and the inner uncontaminated frozen part of the cores was immediately sectioned, placed in Whirl-Pak bags, transported to a mobile –20°C freezer (Waeco CoolFreeze CF-50), and stored frozen until performing genetic analyses. The total community genomic DNA was isolated from ∼5 g of frozen permafrost sample using the PowerSoil kit (Mo Bio Laboratories, Qiagen Company, USA), followed by the Genomic DNA Clean & Concentrator kit (Zymo Research, USA). Metagenome sequencing was performed at the Centre for Genomic Regulation sequencing facility (Barcelona, Spain), employing the Illumina TruSeq SBS kit (v.3) and the Illumina HiSeq 2000 machine with a 2 × 250-cycle sequencing protocol. Metagenome analysis was carried out by first checking the quality of paired reads with Illumina FastQC v.0.11.5 (10) before removing adapters and low-quality reads using Trimmomatic v.0.36 (11), with options LEADING:3 TRAILING:3 SLIDINGWINDOW:4:15 MINLEN:36. Taxonomic classification on trimmed reads was performed with a novel metagenome classifier, Kaiju v.1.7.2 (12), to find matches on the protein level. The reads were compared to the NCBI nr+euk database in greedy mode (minimum match length, 15; minimum match score, 75; and allowed mismatches, 3) (12). Trimmed reads were also individually assembled using MEGAHIT v.1.0.3, with k-mer sizes of 41, 51, 61, 71, 81, and 91 (13). The subsequent assembled contigs were evaluated using QUAST v.5.0.2 (14). Prodigal v.2.6.3 (15) was used to find coding genes on contigs of ≥1,000 bp with a meta flag. The amino acid sequences from predicted genes were subsequently uploaded to GhostKOALA (https://www.kegg.jp/ghostkoala/) for KEGG annotation using the genus_prokaryotes and family_eukaryotes databases (16). After the preprocessing of raw paired reads, we obtained 56 to 77 million trimmed reads per sample, which were assembled into 11,143 to 207,624 contigs of ≥1,000 bp in length, representing 3.81% to 9.42% mapped reads to ≥1,000-bp contigs.

Based upon the taxonomic analysis on trimmed reads, *Actinobacteria* was the most abundant phylum in both the BP (22.4% of reads) and the OR (36.3%) samples, whereas *Proteobacteria* dominated in the GP (17.7%) samples. Archaea were more abundant in the GP samples (3.4% of reads) than in the BP (0.5%) and the OR (1.6%) samples. Of all reads assigned to Archaea in the GP samples, 61% belonged to Euryarchaeota, with which many methanogens are taxonomically affiliated. Genes encoding methyl-coenzyme M reductase (based upon the presence of *mcrABG* genes) that can catalyze the final step of methanogenesis were identified only within the GP samples, which was consistent with the presence of biogenic methane (17). While metagenomes from both the BP and OR samples contained genes assigned to methane/ammonia monooxygenase (based upon the presence of the *pmoABC*-*amoABC* genes), suggesting that microorganisms in these two locations have the potential to oxidize methane to methanol and/or ammonia to hydroxylamine. In addition, enzymes such as endoglucanase (based upon the presence of Enzyme Commission number [EC] 3.2.1.4) and xylan 1,4-beta-xylosidase (based upon the presence of the *xynB* gene) involved in the degradation of complex polysaccharides were identified in all three metagenomes.

Organic-bearing ice-rich Yedoma sediments are vulnerable to environmental changes, including thawing, and these sediments might be considered a potential GHG source in permafrost. A comprehensive investigation of Yedoma metagenomes could provide a basis for more reliable predictions of future biochemical reactions within these sediments in response to climate change.

### Data availability.

The raw sequences for these metagenomes were deposited to the NCBI Sequence Read Archive (SRA) under the accession numbers SRX2163490, SRX2163491, and SRX2163492.
